# Long-Term Effect of Acetylcholinesterase Inhibitors on the Dorsal Attention Network of Alzheimer’s Disease Patients: A Pilot Study Using Resting-State Functional Magnetic Resonance Imaging

**DOI:** 10.3389/fnagi.2022.810206

**Published:** 2022-04-05

**Authors:** Ken-ichiro Yamashita, Taira Uehara, Yoshihide Taniwaki, Shozo Tobimatsu, Jun-ichi Kira

**Affiliations:** ^1^Translational Neuroscience Center, Graduate School of Medicine, International University of Health and Welfare, Otawara, Japan; ^2^Department of Neurology, Brain and Nerve Center, Fukuoka Central Hospital, Fukuoka, Japan; ^3^Department of Neurology, International University of Health and Welfare Narita Hospital, Chiba, Japan; ^4^Department of Neurology, Fukuoka Sanno Hospital, Fukuoka, Japan; ^5^Department of Orthoptics, Faculty of Medicine, Fukuoka International University of Health and Welfare, Fukuoka, Japan

**Keywords:** Alzheimer’s disease, resting-state fMRI, acetylcholinesterase inhibitors, dorsal attention network, resting-state networks

## Abstract

**Background:**

Alzheimer’s disease (AD) is the most common condition of all neurodegenerative diseases and is characterized by various cognitive dysfunctions. Recent resting-state functional magnetic resonance imaging (rs-fMRI) studies have revealed the physiological dynamics of functionally connected brain networks, which are called resting-state networks (RSNs). Associations between impairments of RSNs and various neuropsychiatric diseases, such as AD, have been reported. Acetylcholinesterase inhibitors (AChEIs) have been used as a pharmacological treatment for mild-to-moderate moderate AD, and short-term improvements in cognitive functions and RSNs in restricted areas have been reported.

**Objective:**

We aimed to characterize AChEI-related RSN changes by acquiring two sets of rs-fMRI data separated by approximately 3 to 6 months.

**Methods:**

Seventeen patients with AD and nine healthy subjects participated in this study. Independent component analysis was performed on the rs-fMRI data of AChEI-responsive and non-responsive AD patients, stratified according to change in Mini-Mental State Examination (MMSE) scores after 3 to 6 months of AChEI therapy. In addition, a region of interest-based analysis of the rs-fMRI data before therapy was performed to explore the functional connectivity (FC) changes associated with AchEI therapy.

**Results:**

Responders showed a significantly greater increase in MMSE scores, especially for orientation for time, than that of non-responders following AChEI therapy. A subtraction map of MMSE score differences (responders minus non-responders) in the independent component analysis revealed higher FC of the dorsal attention network in responders compared with that in non-responders. Moreover, in the region of interest analysis of untreated status data, the dorsal attention network showed significant negative FC with the right planum temporale, which belongs to the ventral attention network, proportional to MMSE score change.

**Conclusion:**

The negative correlation of the FC of the dorsal attention network and right planum temporale before AChEI therapy and MMSE score change may be a biomarker of the therapeutic effect of AChEIs for AD.

## Introduction

Alzheimer’s disease (AD) is the most common cause of dementia and presents with a variety of cognitive dysfunctions. In addition to brain atrophy, particularly in the hippocampus and parietal lobe as shown using structural magnetic resonance imaging (MRI) (Frisoni et al., 2010), AD patients exhibit hypoperfusion in the temporal lobe and the medial and lateral parts of the parietal lobe even in the early stage of AD as shown using functional neuroimaging techniques, such as positron emission tomography and single-photon emission computed tomography (Neary et al., 1987; Burns et al., 1989).

Although functional MRI (fMRI) techniques have revealed increased deoxy-hemoglobin during the performance of cognitive tasks in brain regions related to higher brain function (Ojemann et al., 1998), resting-state fMRI (rs-fMRI) studies have demonstrated hyperactivation in the anterior cingulate (AC) gyrus and the medial and lateral areas of the parietal lobe, where amyloid β preferentially deposits when the brain is not engaged in cognitive tasks (Sperling et al., 2009). These regions show synchronized patterns of activation, termed the default mode network (DMN), which is highly active during mind-wandering conditions. Thus, this hyperactivation is assumed to be related to the pathological changes in AD (Busche et al., 2008). Furthermore, rs-fMRI studies have detected several other functionally connected large-scale networks, which are now known as resting-state networks (RSNs) (Yeo et al., 2011; Lee et al., 2012).

Acetylcholinesterase inhibitors (AChEIs) and N-methyl-D-aspartate receptor antagonists (memantine) were developed according to the amyloid cascade hypothesis and are currently used as a therapeutic approach for AD (Özbey et al., 2016; Özgeriş et al., 2016). These drugs are effective in improving the cognitive functions and psychological symptoms of patients with AD (Rogers et al., 1998; Reisberg et al., 2003). A double-blind placebo-controlled study of AD patients revealed a significantly greater improvement in the cognitive component score of the Alzheimer’s Disease Assessment Scale Japanese version (ADAS-J cog) for 24 weeks in the donepezil-treated group compared with that in the placebo group (Homma et al., 2000). Furthermore, a randomized comparison study of donepezil and galantamine in AD patients indicated that the long-term efficacy of AChEIs on Mini-Mental State Examination (MMSE) score lasted at least 26 weeks; moreover, in patients who received galantamine, MMSE scores at 52 weeks were not significantly different from those at baseline (Wilcock et al., 2003).

An rs-fMRI study that evaluated the association between AChEI effects and functional connectivity (FC) changes in AD patients reported increased FC in the orbitofrontal network, which correlated with cognitive improvement after 12 weeks of treatment (Griffanti et al., 2016). Although the increased FC of the orbitofrontal network with AChEI treatment based on independent component analysis (ICA) reflects the improvement in cognitive function in AD patients, FC differences in RSNs between responders and non-responders before AChEI therapy has not yet been explored.

Therefore, in this study, we aimed to examine FC changes associated with AChEI therapy for AD by acquiring rs-fMRI data at two time points: before and after AChEI treatment. We performed a correlation analysis between cognitive scale scores (MMSE and its subscale scores) and FC among the RSNs of the rs-fMRI data acquired before AChEI therapy.

## Materials and Methods

### Subjects

Seventeen AD patients (six males and 11 females; mean age ± standard deviation [SD], 77.3 ± 8.2 years) participated in the study from 2015 to 2019 at Fukuoka Sanno Hospital, and nine age-matched healthy controls (three males and six females; aged 71.5 ± 11.5 years) were enrolled in the study. Diagnosis of AD was made by two neurologists (KY and YT), based on National Institute on Aging and Alzheimer’s Association criteria (McKhann et al., 2011). Brain MRIs of all subjects showed no abnormalities except brain atrophy. Subjects were all right-handed and had no history of neurological disorders. None of the subjects were taking psychiatric medications, including memantine.

### Procedure

**FIGURE 1:**
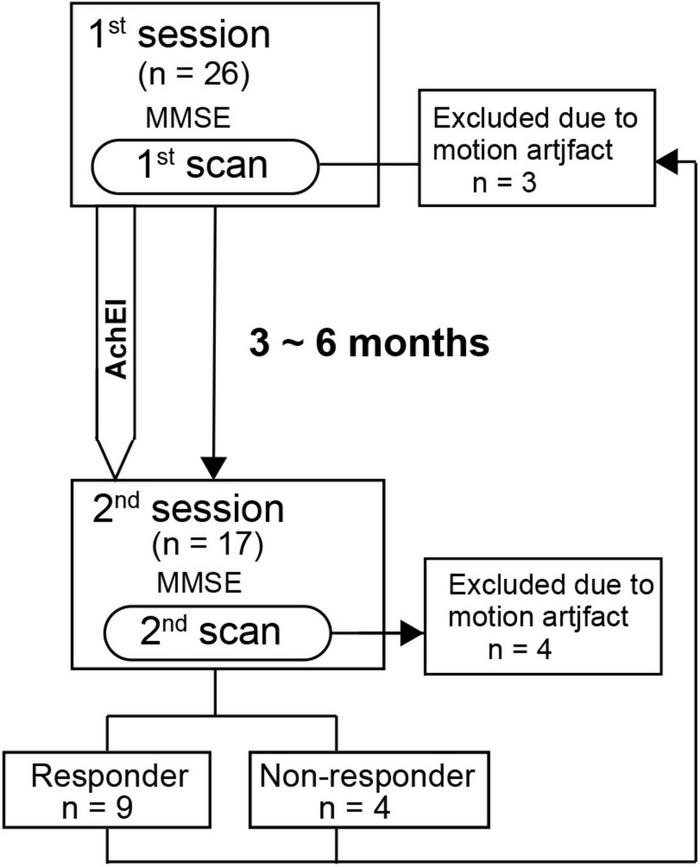
Time schedule for the detection of resting-state network changes following acetylcholinesterase inhibitor therapy in patients with Alzheimer’s disease.

All subjects except normal subjects participated in two sessions, each of which comprised the MMSE (Folstein et al., 1975) and an rs-fMRI scan. The first and second sessions were separated by approximately 3 to 6 months, which corresponds to the duration of AChEI effectiveness as shown in previous studies (Figure 1). AChEIs indicate the effect of acetylcholine (ACH) increment by inhibiting acetylcholinesterase (AchE). AChE activity was measured using a spectrophotometer, which estimates colorimetric product (412 nm) formed from a reaction between 5,5′-dithgiobis (2-nitrobenzoic acid) and AChE-derived thiocholine (Özgeriş et al., 2016). During the first session, the MMSE was administered to all participants, and the main subscale scores of orientation for time (OT, scale 0–5), orientation for place (scale 0–5), delayed recall (DR, scale 0–3), and calculation (scale 0–5) were used for subsequent analyses. After the first rs-fMRI scan, AChEI therapy was initiated for AD patients (eight received donepezil, seven received rivastigmine, and two received galantamine). Although various criteria of increased MMSE score have been used previously (Miranda et al., 2015; Gallucci et al., 2016), we classified patients into responders and non-responders during the second session according to criteria based on the Mendiondo model-estimated curve of MMSE decline using a cubic or logarithmic function of MMSE score (Mendiondo et al., 2000). Briefly, the responder group consisted of subjects with second session MMSE scores that were higher than estimated MMSE scores based on the Mendiondo model, and the non-responder group consisted of subjects with MMSE scores that were lower than those estimated by the model. A Mann-Whitney test was used to compare the demographic features and cognitive performance between the two groups.

### Image Acquisition

A 3 Tesla MRI scanner (Achieva TX, Philips Medical Systems, Netherlands) was used to acquire MRI data during both study sessions. Scout images were first collected to center the field of view on the subject’s brain. Three dimensional T1-weighted turbo field echo images were obtained for anatomical reference (repetition time = 8.1 ms; echo time = 3.8 ms; field of view = 240 mm; matrix size = 240 × 240; slice thickness = 1.0 mm). For functional imaging, a gradient-echo echo-planar sequence was used (repetition time = 2,800 ms; echo time = 30 ms; flip angle = 90 degrees; 40 × 3 mm slices; in-plane resolution of 3 × 3 mm). One functional run was acquired, and the total scanning time was 7 min.

### Image Analysis

Preprocessing of imaging data was performed using the SPM8 software^1^. Functional images were slice timing corrected, realigned, normalized to echo-planar imaging template based on Montreal Neurological Institute coordinates, and spatially smoothed (full width at half maximum = 8 mm). The first five functional volumes were excluded from the analysis to take into account the equilibrium of longitudinal magnetization.

To reduce motion-related effects during rs-fMRI scanning, we calculated framewise displacement, which represents a six-dimensional time series across frames for each subject (Power et al., 2012). Subjects who had more than 90% of time points with a framewise displacement below 0.5 mm were included for analysis (Power et al., 2014). This criterion was applied to both the first and second scans.

In the first step, we performed ICA on the second scan data using the CONN software^2^ (Whitfield-Gabrieli and Nieto-Castanon, 2012). The gray matter signal was filtered using a bandpass filter (0.01–0.1 Hz) to reduce the effect of low-frequency physiologic noise. White matter and cerebrospinal fluid signals were regressed out during the denoising step. The group ICA analysis using the fast ICA algorithm involved concatenating the blood-oxygen-level-dependent signal across subjects to identify independent spatial components and back-projecting these components onto individual subjects, which resulted in regression coefficients maps that represented the FC between the independent components (Calhoun et al., 2001). We set 20 independent components to be identified, which included the DMN and the sensorimotor, frontoparietal, dorsal attention (DAN), salience (SN), language, and visual networks. We then generated a subtraction map of the drug efficacy differences between the responder and non-responder groups in each session to identify FC changes due to AChEI treatment.

After performing ICA of the second scan data, we conducted a region of interest (ROI) analysis, whereby ROIs were anatomically defined using the FSL Harvard-Oxford maximum probability cortical atlas, with bilateral regions divided into left and right hemispheres (168 ROIs) in the first scan data. The anatomical regions and RSNs that were significantly different between responders’ and non-responders’ second scan data were plotted as seeds for the ROI-to-ROI analysis of the first scan data. Using the same data for seed selection and ROI analysis results in distorted descriptive statistics and invalid statistical inferences (Kriegeskorte et al., 2009). Moreover, several studies have performed ICA in one group with no *a priori* assumptions for deciding the seed for a subsequent ROI analysis of another group (Brier et al., 2012; Tsvetanov et al., 2016). Therefore, we performed ICA on the second scan data to establish the seed and subsequently performed ROI-to-ROI analysis using the first scan data. Pearson linear correlation coefficients between the time course of the signal of each ROI and the average signal of the seed were calculated using CONN. ROI-to-ROI one-sample *t*-tests were used to determine significant connectivity between the seed and ROI in each group. Connectivity estimated by the ICA was considered significant when the corrected *p*-value for the cluster-level false-discovery rate (*p*-FDR) was less than 0.05 (Bueno et al., 2018). Seed-level *p*-FDR as implemented in CONN was used for the statistical analysis of the ROI analysis. The threshold used for the seed-level *p*-FDR was set at 0.05.

Spearman’s rank-order correlation analysis was performed in the AD patients to verify the association between MMSE subscale score change from the first to the second session and z-transformed *r*-values of the seed to significant ROI for the responders versus non-responders contrast.

## Results

### Behavior Data

All patients completed at least 3 months of AChEI therapy (mean doses of donepezil, galantamine, and rivastigmine were 6, 16, and 10.5 mg/day, respectively). Second scan data of four subjects (three responders and one non-responder) and first scan data of three subjects (three responders) were excluded because of excessive FD criteria. The ratio of time points with FD below 0.5 mm in the rs-fMRI scan of the responder, non-responder, and healthy control groups were 92.8 ± 5.9%, 97.8 ± 2.8%, and 97.4 ± 3.4% in the first session, respectively. The FD values of the responders and non-responders were 95.9 ± 2.1% and 96.7 ± 2.5% in the second session, respectively. Finally, the second scan data of 13 subjects (nine responders and four non-responders) and the first scan data of 19 subjects (six responders, four non-responders, and nine healthy subjects) were included in the final analyses. There were no significant differences in age at examination among the three groups. In addition, the interval between the first and second scans did not differ significantly between the responder group (mean ± SD: 144.9 ± 43.5 days) and non-responder groups (mean ± SD: 132.5 ± 46.8 days; i.e., the therapeutic duration did not significantly differ between the two groups).

The MMSE scores of the responders, non-responders, and healthy control groups were 18.4 ± 2.6, 19.0 ± 1.2, and 28.7 ± 1.6 for the first session, respectively, and 22.1 ± 2.5 and 17.5 ± 2.4 for the second session (healthy controls completed the MMSE during the first session only), respectively. In the responder group, three received donepezil, four received rivastigmine, and two received galantamine. In the non-responder group, two received donepezil and two received rivastigmine. All main MMSE subscale scores of the first session were not significantly different between responders and non-responders; however, both MMSE (*p* < 0.01) and main MMSE subscale scores (OT, orientation for place, delayed recall, and calculation) were significantly lower in patients than in healthy controls. Compared with responders, non-responders had significantly lower MMSE (*p* < 0.05) and OT (*p* < 0.05) scores in the second session (Table 1).

**TABLE 1:** Demographic features of healthy controls, responders, and non-responders.

	AD		Normal
	**Responder**	**Non-responder**	
		
*N*	9	4	9
Age	76.2 ± 10.6	74.8 ± 3.0	71.5 ± 11.3
M:F	3:6	2:2	3:6
Days (Scan 1 – Scan 2)	144.9 ± 43.5	132.5 ± 46.8	
MMSE (1st)	18.4 ± 2.6^††^	19.0 ± 1.2^††^	28.7 ± 1.6
OT (0–5)	2.4 ± 0.9^††^	1.8 ± 0.5^††^	4.6 ± 0.4
OP (0–5)	3.3 ± 1.0^††^	3.0 ± 1.2^††^	5.0 ± 0.0
DR (0–3)	1.0 ± 0.9^†^	0.8 ± 1.0^†^	2.4 ± 0.7
Calculation (0–5)	1.7 ± 1.3^††^	2.8 ± 1.5^†^	4.6 ± 0.7
MMSE (2nd)	22.1 ± 2.5	17.5 ± 2.4*	
OT (0–5)	3.2 ± 1.2	1.3 ± 1.3*	
OP (0–5)	4.3 ± 0.7	2.8 ± 1.7	
DR (0–3)	0.9 ± 0.8	0.5 ± 1.0	
Calculation (0–5)	2.9 ± 1.4	2.8 ± 1.5	

Data are shown as means ± standard deviations. The first MMSE test was performed on six responders and four non-responders. ^†^p < 0.05 compared with healthy subjects. ^††^p < 0.01 compared with healthy subjects. *p < 0.05 compared with responders. MMSE, Mini-Mental State Examination; OP, orientation for place; OT, orientation for time; DR, delayed recall.

### Imaging Data

**FIGURE 2:**
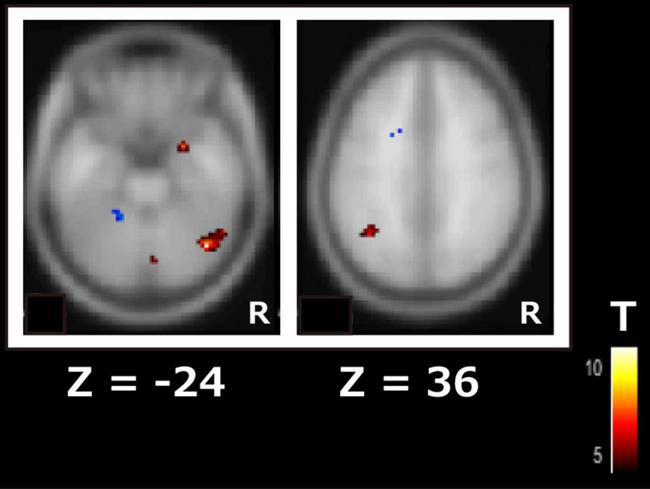
Subtraction map of responders minus non-responders for the dorsal attention network using independent component analysis of the second scan data. The color bar indicates the *t*-value.

Among the RSNs related to cognitive function changes, the ICA of the second session revealed significant differences in drug efficacy between responders and non-responders in the DAN and visual network. In the DAN, the right inferior temporal gyrus (*t* = 11.2, p-FDR < 0.05) and left angular gyrus (*t* = 6.9, p-FDR < 0.05) showed significantly higher FC in responders rather than non-responders (Figure 2). In the visual network, the left cerebellum (*t* = 7.6, *p*-FDR < 0.05) had significantly higher FC in the responders than in the non-responders.

**FIGURE 3:**
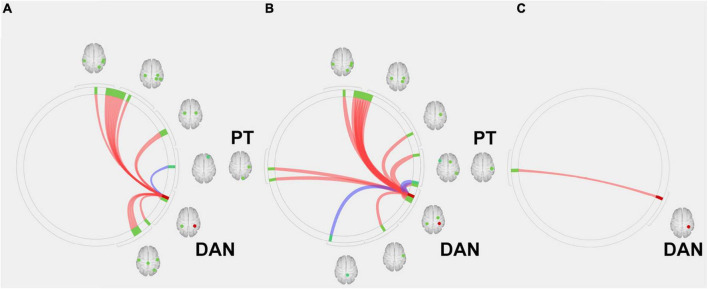
Region of interest (ROI)-to-ROI connectivity values for regions significantly (*p* < 0.05 false-discovery rate corrected) connected with the dorsal attention network in **(A)** healthy subjects, **(B)** responders, and **(C)** and non-responders. Red and blue lines indicate positive and negative connectivity, respectively. Red circles indicate the seed (DAN), and green circles indicate significant ROIs. DAN, dorsal attention network; PT, planum temporale.

In the next step, we used the DAN, which is related to higher cognitive functions, as seeds (containing 3,137 voxels) for the ROI-to-ROI analysis of the first scan dataset before AChEI therapy. In healthy subjects, the DAN was significantly connected to the bilateral superior parietal lobules, bilateral pre- and postcentral gyri, bilateral supramarginal gyri, right inferior temporal gyrus, and right lateral occipital cortex (Figure 3A). Although responders showed significant FC between the DAN and right planum temporale (PT) and the primary sensory and motor areas, similarly to healthy subjects (Figure 3B), only the FC between the DAN and right PT was significant in non-responders (Figure 3C and Table 2).

**TABLE 2:** Regions significantly connected with the dorsal attention network in the region of interest analysis.

	Region	T	p-FDR
Normal	Rt. Superior Parietal Lobule	17.75	0.0001
	Rt. Postcentral Gyrus	13.80	0.0001
	Rt. Supramarginal Gyrus	9.46	0.0007
	Lt. Postcentral Gyrus	6.57	0.0048
	Rt. Precentral Gyrus	6.32	0.0053
	Lt. Supramarginal Gyrus	6.09	0.0053
	Lt. Superior Parietal Lobule	6.02	0.0053
	Rt. Frontal Pole	−5.56	0.0053
	Lt. Precentral Gyrus	5.10	0.0119
	Rt. Inferior Temporal Gyrus	4.87	0.0147
	Rt. Lateral Occipital Cortex	3.90	0.0450
Responder	Lt. Superior Parietal Lobule	12.22	0.0070
	Rt. Supramarginal Gyrus	10.88	0.0070
	Rt. Superior Parietal Lobule	9.23	0.0076
	Rt. Superior Frontal Gyrus	9.07	0.0076
	Rt. Lateral Occipital Cortex	8.22	0.0091
	Rt. Occipital Pole	6.24	0.0225
	Rt. Precentral Gyrus	5.99	0.0225
	Rt. Postcentral Gyrus	5.96	0.0225
	Rt. Planum Temporale	5.93	0.0225
	Rt. Cerebellum	−5.88	0.0225
	Lt. Supramarginal Gyrus	5.07	0.0405
	Lt. Postcentral Gyrus	4.81	0.0475
Non-responder	Rt. Planum Temporale	24.02	0.0264
Responder > Non-responder	Rt. Planum Temporale	−6.18	0.0441

**FIGURE 4:**
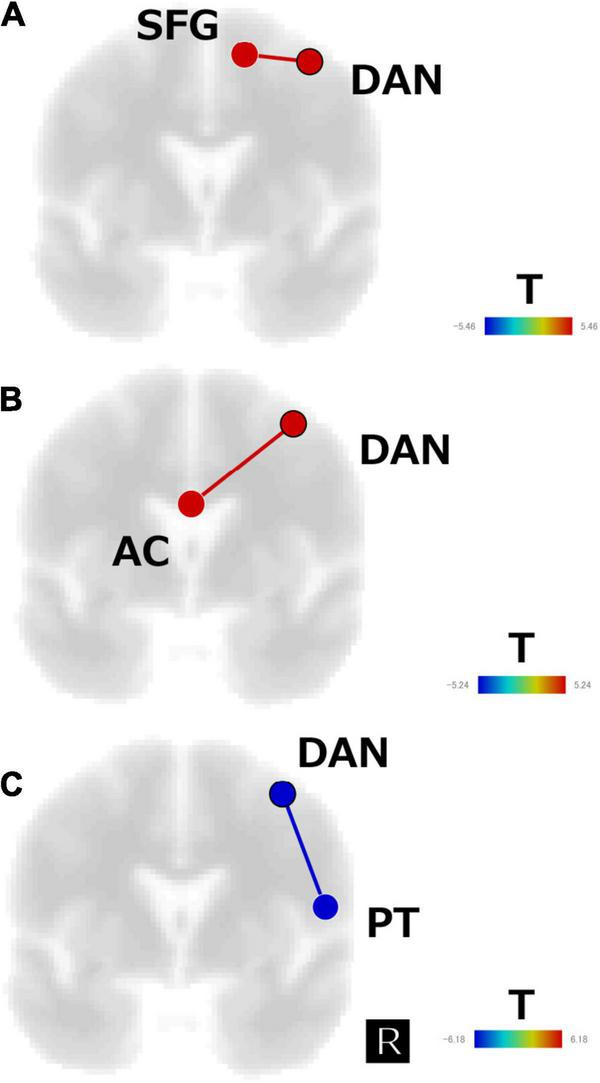
Subtraction map of **(A)** responders minus healthy subjects, **(B)** non-responders minus healthy subjects, and **(C)** responders minus non-responders for the regions of interest analysis of the first scan data. The connectivity map is displayed in the coronal view and overlaid on a template image. The color bar indicates the *t*-value. SFG, superior frontal gyrus; AC, anterior cingulate; DAN, dorsal attention network; PT, planum temporale.

The subtraction z-score map of responders versus healthy control subjects revealed significant positive FC between the DAN and the right superior frontal gyrus (SFG) (*t*[13] = 5.5, *p* < 0.05, FDR corrected; Figure 4A), whereas the subtraction z-score map of non-responders versus healthy controls showed significant positive FC between the DAN and AC (*t*[11] = 5.2, *p* < 0.05, FDR corrected; Figure 4B). Responders showed significant negative FC between the DAN and right PT (*t*[8] = −6.2, *p* < 0.05, FDR corrected; Figure 4C), which was not observed in the non-responders.

For elucidating the differences among three AChEIs, we conducted the ROI analysis using the first scan data of the five AD patients (three responders and two non-responders) who used donepezil. As a result, no significant regions detected in the contrast of responders versus non-responders were related to donepezil. ROI analysis of patients treated with rivastigmine or galantamine was not performed because of the small sample size (only three patients were treated with rivastigmine, and two patients were treated with galantamine).

To examine the relationship between AchEI treatment on cognitive function and FC before therapy, we performed Spearman’s correlation analysis between change in MMSE score (ΔMMSE; second session MMSE score minus first session MMSE score)/first session MMSE score and z-transformed *r*-values from the DAN to the right SFG, AC, and right PT ROIs that were significantly connected to the DAN as a therapeutic index. Similarly Spearman’s correlation analysis between change in OT score (ΔOT; second session OT score minus first session OT score)/first session OT score and z-transformed *r*-values from the DAN to the right SFG, AC, and right PT was executed.

**FIGURE 5:**
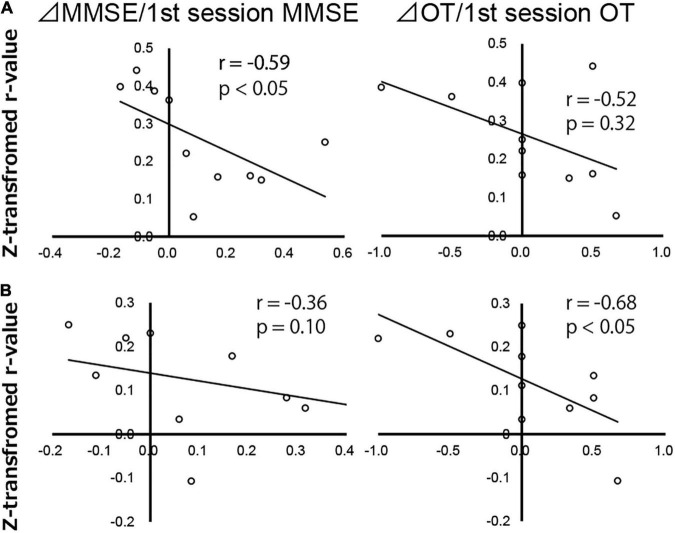
Correlation map of Alzheimer’s disease patients between change in Mini-Mental State Examination score (ΔMMSE)/first session MMSE score, change in orientation for time score (ΔOT)/first session OT score, and z-transformed *r*-values of the dorsal attention network to the **(A)** right planum temporale and **(B)** anterior cingulate gyrus, analyzed using Spearman’s rank correlation test. Each circle represents data from one subject.

Results revealed no significant correlation between ΔOT/first session OT, ΔMMSE/first session MMSE scores, and z-transformed *r*-values from the DAN to the right SFG. Although the FC between the DAN and right PT was significantly negatively correlated with ΔMMSE/first session MMSE score (*r* = −0.59, *p* < 0.05), there was no significant correlation between the DAN and right PT FC and ΔOT/first session OT score (Figure 5A). The FC between the DAN and AC was negatively correlated with ΔOT/first session OT score (*r* = −0.68, *p* < 0.05). There was no significant correlation between ΔMMSE/first session MMSE score and FC between the DAN and AC (Figure 5B).

## Discussion

In the present study, we revealed that compared with AD patients who did not respond to AChEIs, those who did respond exhibited lower FC between the DAN and right PT (part of the ventral attention network [VAN]) before therapy. This suggested that the FC between the DAN and VAN is associated with the decline in MMSE scores in AchEI-resistant AD patients. Moreover, AD patients who did not respond to AChEIs showed significantly greater positive FC between the DAN and AC than that of normal subjects; moreover, a significant OT-related decrement in *r*-values was revealed in these areas.

Anatomically, the PT is part of the superior temporal gyrus, and the left PT is associated with verbal processing (Hickock, 2009). The significantly higher FC between the DAN and right PT pre-AchEI therapy differentiated non-responders from responders (Corbetta and Shulman, 2002; Hirnstein et al., 2013). However, AchEI-responsive AD patients maintained significant FC from the DAN to the primary sensory and motor areas, similar to that of healthy subjects, except for the FC to the right PT.

A previous single-photon emission computed tomography study of AD patients who were non-responsive to donepezil showed a significantly greater reduction in regional cerebral blood flow of the lateral and medial frontal lobes compared with that of responders (Hanyu et al., 2003). Moreover, several rs-fMRI studies in Apolipoprotein E ε4-positive AD patients reported higher FC of the bilateral dorsolateral prefrontal cortex (part of the DAN) following donepezil therapy (Zaidel et al., 2012) and higher FC of the DAN, the control network, and SN after AchEI treatment than that of untreated AD patients (Wang et al., 2014). These AchEI-related FC increases in the attentional network are consistent with our ICA results following AchEI treatment and suggest that improvement in attentional function may ameliorate the OT score.

Although several rs-fMRI studies have investigated the effects of AChEIs, few studies have focused on the FC between RSNs before therapy. One rs-fMRI study directly compared imaging data before and after AchEI therapy using the middle and posterior cingulate cortices as seeds and found significant increases in the FC of the parahippocampal, temporal, parietal, and prefrontal cortices, which largely overlap the DMN (Li et al., 2012). Another rs-fMRI study that directly compared FC between before and 12 weeks after donepezil treatment reported increased FC in the orbitofrontal network with improvement in cognitive function (Griffanti et al., 2016). In contrast, our ICA on rs-fMRI data did not reveal any significant differences in FC, other than in the DAN, between responders and non-responders after long-term AchEI therapy.

Both AD patients who were responsive and non-responsive to AchEI treatment showed higher FC between the PT and the DAN. Moreover, the ROI analysis showed that compared with healthy control subjects, patients showed higher FC between the SFG and AC and the DAN. Previous studies on the inter-RSN FC in AD patients reported higher FC between the frontoparietal network and the DMN in AD patients than in cognitively normal subjects (Contreras et al., 2019). Furthermore, increases in the FC between the DMN and the DAN similar to those observed in our study have been demonstrated in studies comparing AD patients and individuals with mild cognitive impairment (Wang et al., 2019). In contrast to the findings on inter-RSN FC, AD patients have been found to have lower intra-RSN FC than that of MCI and healthy subjects (Zhang et al., 2015). More specifically, AD patients have shown lower intrinsic FC in the memory-related subsystems of the DMN (Qi et al., 2018). Therefore, the functional separation of RSNs from the DAN following AchEI treatment may underlie the improvement in MMSE scores, especially OT scores.

In addition to AD patients, an rs-fMRI study using ICA reported higher FC between the DMN and the central executive network in patients with schizophrenia than in healthy controls, and the severity of hallucinations was positively correlated with the DMN–CEN FC (Manoliu et al., 2014a). Furthermore, patients with major depressive disorder show higher inter-RSN FC between the SN and DMN, coupled with lower intra-FC within the anterior insula, which belongs to the SN, than do healthy controls (Manoliu et al., 2014b). Thus, the variability in FC, especially the hyperconnectivity between RSNs, may underlie the pathophysiology of various neuropsychiatric diseases.

A previous rs-fMRI study revealed that OT score positively correlates with FC between the VAN and posterior cingulate cortex, which belongs to the DMN (Yamashita et al., 2019). Responders in our study showed higher OT scores alongside lower FC between the DAN and PT, which is near the VAN, compared with non-responders, whereas non-responders exhibited higher FC between the DAN and AC gyrus, which belongs to the DMN. Therefore, lower FC between the DAN and VAN-DMN combination may induce future improvements in the OT score. Thus, an rs-fMRI scan in AD patients before therapy may predict the therapeutic effect of AChEIs.

A limitation of this study is the relatively small sample size due to the exclusion of patients because of motion artifacts. Therefore, our results should be considered preliminary and require confirmation in a larger sample size study. The effect of white matter burden and APOE4 as representative confounders of the fMRI studies especially in AD patients were not estimated in this study. It should be expected removing these potential confounders in the future studies. Although we presumed there was no significant difference of brain atrophy between responders and non-responders based on visual inspection of structural MR images, measurement of brain atrophy should be performed like former confounders.

In addition, several prospective studies with pre-therapeutic rs-fMRI scans are necessary to confirm the effects of AChEIs on the DAN. Because the increase in intra-network FC of the DMN was reported after 12 months of galantamine use (Blautzik et al., 2016), intra-network FC analyses of the DAN, VAN, and DMN before and after therapy should be performed in addition to inter-network FC analyses. Nevertheless, despite these limitations, the functional dysconnection between the DAN and VAN observed in this study may be a potential therapeutic biomarker for AD.

## Conclusion

Improvement in MMSE scores, particularly OT subscale scores, in AChEI-responsive AD patients was associated with increased FC of the DAN. ROI analysis using a DAN seed of rs-fMRI data before therapy revealed that FC changes between the right PT and AC were negatively correlated with MMSE and OT score changes. These results suggested that the increased FC between RSNs may be involved in the cognitive dysfunctions observed in patients with AD.

## Data Availability Statement

The raw data supporting the conclusions of this article will be made available by the authors, without undue reservation.

## Ethics Statement

The studies involving human participants were reviewed and approved by the Fukuoka Sanno Hospital. The patients/participants provided their written informed consent to participate in this study.

## Author Contributions

K-IY: conceptualization and data analysis. TU: data analysis. YT: data collection. ST and J-IK: supervise the research. All authors contributed to the article and approved the submitted version.

## Conflict of Interest

The authors declare that the research was conducted in the absence of any commercial or financial relationships that could be construed as a potential conflict of interest.

## Publisher’s Note

All claims expressed in this article are solely those of the authors and do not necessarily represent those of their affiliated organizations, or those of the publisher, the editors and the reviewers. Any product that may be evaluated in this article, or claim that may be made by its manufacturer, is not guaranteed or endorsed by the publisher.

## Footnotes

1
http://www.fil.ion.ucl.ac.uk/spm/

2
https://www.nitrc.org/projects/conn/
